# Serological survey of canine H3N2, pandemic H1N1/09, and human seasonal H3N2 influenza viruses in cats in northern China, 2010–2014

**DOI:** 10.1186/s12985-015-0285-5

**Published:** 2015-04-01

**Authors:** Xuxiao Zhang, Ye Shen, Lijie Du, Ran Wang, Bo Jiang, Honglei Sun, Juan Pu, Degui Lin, Ming Wang, Jinhua Liu, Yipeng Sun

**Affiliations:** State Key Laboratory of Agrobiotechnology, College of Veterinary Medicine and Key Laboratory of Animal Epidemiology and Zoonosis of the Ministry of Agriculture, China Agricultural University, No. 2 Yuanmingyuan West Road, Beijing, 100193 China

**Keywords:** Influenza, Antibodies, Cat

## Abstract

**Background:**

The close contact between cats and humans poses a threat to public health because of the potential zoonotic transmission of influenza viruses to humans. Therefore, we examined the seroprevalence of pandemic H1N1/09, canine H3N2, and human H3N2 viruses in pet cats in northern China from 2010 to 2014.

**Finding:**

Of 1794 serum samples, the seropositivity rates for H1N1/09, canine H3N2, and human H3N2 were 5.7%, 0.7%, and 0.4%, respectively. The seropositivity rate for H1N1/09 in cats was highest in 2010 (8.3%), and then declined continuously thereafter. Cats older than 10 years were most commonly seropositive for the H1N1/09 virus.

**Conclusions:**

Our findings emphasize the need for continuous surveillance of influenza viruses in cats in China.

Different subtypes of influenza viruses are reported to be naturally transmitted to cats from other species worldwide, including avian viruses (H5N1), canine viruses (H3N2), and human viruses (pandemic H1N1/09, seasonal H1N1, and H3N2) [1-3]. The close contact between cats and humans possesses a threat to public health because of the potential zoonotic transmission of influenza viruses to humans. In China, the canine H3N2 and H1N1/09 influenza viruses circulate in dogs [4], and the seasonal H3N2 and H1N1/09 viruses are prevalent in humans [5,6], any of which might be transmitted to cats. Therefore, we examined the seroprevalence of the pandemic H1N1/09, canine H3N2, and human seasonal H3N2 influenza viruses in cats in northern China from January 2010 to June 2014.

A total of 1794 serum samples were collected from domestic cats that presented to the Veterinary Teaching Hospital of China Agricultural University between January 2010 and June 2014 (average, 33.2 ± 9.7 samples/month). These cats were from Beijing, Tianjin, Hebei, Henan, and Shandong Provinces. The A/canine/Beijing/359/2009 (H3N2) canine influenza virus has been described previously [7]. The pandemic A/Beijing/7/2009 (H1N1) and seasonal A/Beijing/126/2012 (H3N2) viruses were isolated from patients with influenza-like illnesses. These viruses were used for both hemagglutination inhibition (HI) and microneutralization (MN) assays. The serum samples were treated with receptor-destroying enzyme (RDE; 1 part serum, 3 parts RDE; Denka Seiken, Ltd, Tokyo, Japan) for 18 h at 37°C, which was then heat inactivated at 56°C for 30 min. Then, 25 μL of serial two fold dilutions of the treated serum samples were mixed with 4 HA units of virus in V-shaped microtiter plates and incubated at room temperature for 30 min. Next 25 μL of 1% chicken RBCs was added to each well and incubated at room temperature for 30 min [8]. The HI titers of tested serum samples ranged from 0 to 512, and samples with HI antibody titers ≥ 32 were considered positive. HI antibody titer is defined as the reciprocal of the highest serum dilution that completely inhibits hemagglutination reaction. WHO guidelines for vaccine evaluation suggest that HI antibody titers 40 indicate 50% protection against influenza A virus [9,10]. Therefore, we defined HI antibody titers 32 as the cut-off levels to estimate the infection rates [11,12]. Of the tested serum samples, 102 (5.7%), 12 (0.7%), and 8 (0.4%) were positive for H1N1/09, canine H3N2, and human H3N2 influenza-virus-specific antibodies, respectively. The seropositivity rate for H1N1/09 in cats was highest in 2010 (8.3%), and then declined continuously thereafter (Figure 1), whereas those for the canine H3N2 and human H3N2 viruses were relatively low in each year. The geometric mean titers (GMTs) of the antibodies against the H1N1/09, canine H3N2, and human H3N2 viruses were 139.9, 149.3, and 44.0, respectively (Figure 2).

**Figure 1:**
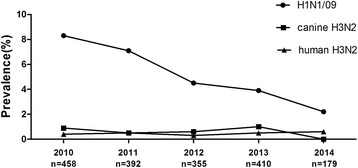
**Yearly seroprevalence of influenza viruses in domestic cats in northern China.**

**Figure 2:**
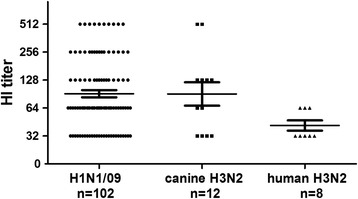
**HI titers in serum samples positive for H1N1/09, canine H3N2, or human H3N2 virus in cats.** In total, 1794 serum specimens were tested with an HI assay, and HI antibody titers ≥ 32 were considered positive. The number of positive serum samples for each virus is shown. GMTs and 95% confidence intervals are indicated by long and short horizontal lines, respectively.

All the serum samples that were HI-positive for H1N1/09, canine H3N2, or human H3N2 viruses and a negative control serum sample were tested for each of the three viruses with an enzyme-linked immunosorbent assay (ELISA)-based MN assay [13]. Briefly, the treated sera were serially diluted two fold in duplicate and incubated with the test virus at a final viral concentration of 100 50% tissue culture infection dose (TCID_50_)/100 μL for 2 h at 37°C. The serum–virus mixture was transferred onto an MDCK cell monolayer maintained in minimum essential medium supplemented with TPCK trypsin (2 μg/mL; Worthington Biochemical Corp., USA) and incubated for 24 h. The reaction plate of the MN assay was tested with an ELISA for the presence of the viral nucleoprotein using a specific mouse monoclonal antibody (AA5H; 1:1500 dilution; Abcam Ltd, Hong Kong) as the primary antibody and a goat anti-mouse IgGs antibody as the secondary antibody. The antibody titer was defined as the reciprocal of the highest serum dilution that reduced the amount of viral nucleoprotein in the reaction wells by 50% compared with that in the control wells. The MN titers for the tested strains in the selected samples were all ≥ 80 (range, 80–1280), which correlated with the HI titers according to Spearman’s rank correlation (correlation coefficient = 0.309; *P* = 0.001), and minimal nonspecific cross-reactivity (MN titers ≤ 20) was observed among the subtypes (Figure 3).

**Figure 3:**
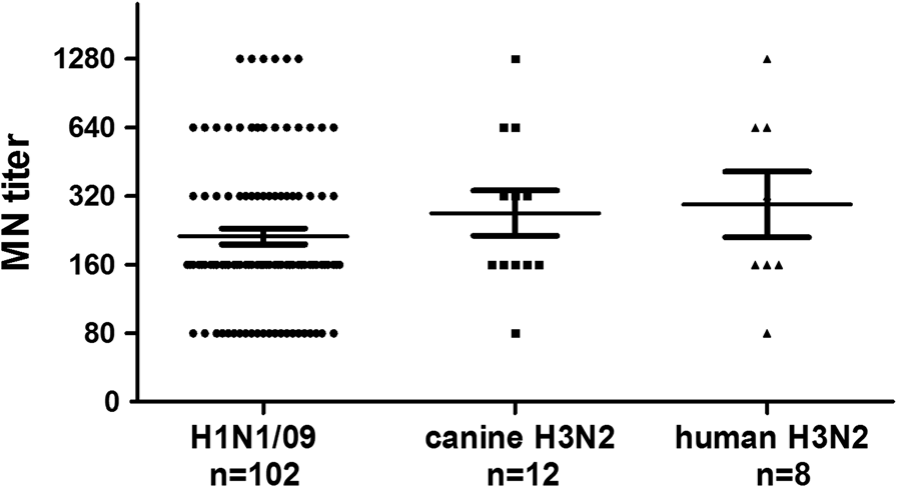
**MN titers in serum samples positive for H1N1/09, canine H3N2, or human H3N2 virus in cats.** The controls and nonspecific cross-reactivity samples (MN titers ≤ 20) were included.

The associations between age, sex or seasonality and the seroprevalence of H1N1/09, canine H3N2, and human H3N2 in cats were investigated. Pearson’s χ^2^ test was used to compare the differences between groups. The results are presented in Table 1, and showed that the seroprevalence of H1N1/09 differed statistically significantly among the different age groups (*P* < 0.001). The seropositive rate for H1N1/09 was higher in cats aged more than 10 years than in the other age groups. There were no statistically significant differences in the seroprevalence of either the canine H3N2 (*P* = 0.876) or human H3N2 (*P* = 0.754) virus among the age groups. No significant differences in the seroprevalence of any virus tested according to sex or seasonality were observed (*P* > 0.05).

**Table 1:** **Characteristics of the tested cats for three influenza viruses and the seroprevalence of the sera against these influenza viruses**

**Characteristic**	**No. (%) samples** ^**a**^	**No. (%) positive**		
		**H1N1/09** ^**b**^	**Canine H3N2** ^**c**^	**Human H3N2** ^**d**^
*Age* (*year*)				
≤2	455 (25.4%)	12 (2.6%)	2 (0.4%)	1 (0.2%)
3-6	463 (25.8%)	13 (2.8%)	4 (0.9%)	3 (0.6%)
7-9	334 (18.6%)	21 (6.3%)	2 (0.6%)	2 (0.6%)
≥10	542 (30.2%)	56 (10.3%)	4 (0.7%)	2 (0.4%)
		*P* ^e^ < 0.001	*P* = 0.876	*P* = 0.754
*Sex*				
Male	1028 (57.3%)	53 (5.2%)	5 (0.5%)	4 (0.4%)
Female	766 (42.7%)	49 (6.4%)	7 (0.9%)	4 (0.5%)
		*P* = 0.261	*P* = 0.272	*P* = 0.676
*Season*				
Spring (Mar–May)	578 (32.2%)	39 (6.7%)	2 (0.3%)	1 (0.2%)
Summer (Jun–Aug)	352 (19.6%)	15 (4.3%)	3 (0.9%)	1 (0.3%)
Autumn (Sep–Nov)	446 (24.9%)	22 (4.9%)	4 (0.9%)	4 (0.9%)
Winter (Dec–Feb)	418 (23.3%)	26 (6.2%)	3 (0.7%)	2 (0.5%)
		*P* = 0.356	*P* = 0.694	*P* = 0.357

^a^HI positive samples were analyzed. ^b^H1N1/09 is A/Beijing/7/2009 (H1N1). ^c^Canine H3N2 is A/canine/Beijing/359/2009 (H3N2). ^d^Human H3N2 is A/Beijing/126/2012 (H3N2). ^e^Pearson’s χ^2^ test was used to compare the differences between groups.

Although neither H1N1/09, canine H3N2, nor human H3N2 has previously been isolated from cats in China, we provided serological evidence that these viruses infected cats in this country. H1N1/09 influenza virus has been repeatedly detected in cats in other countries, and the infected cats present severe respiratory symptoms or even death [14-16]. Evidence of the cat-to-cat transmission of H1N1/09 has be observed both experimentally and clinically [15,17]. In contrast, only one canine H3N2 strain has been isolated from cats [2], and no human seasonal H3N2 virus has been detected in cats. Consistent with these data, the seroprevalence of H1N1/09 (5.7%) was higher than that of canine H3N2 (0.7%) or human seasonal H3N2 (0.4%) in the present study.

Cats aged 10 years or more had a higher seroprevalence of H1N1/09 influenza virus than the other age groups, which might be attributable to the reduced immunity of old cats. The seropositivity rate of H1N1/09 influenza in this study (5.7%) was higher than that in domestic cats in Germany in the period from 2010 to 2011 (1.9%) [18], or in cats in southern China (1.2% by ELISA and 0.6% by HI) [19]. A high prevalence of H1N1/09 was observed among domestic cats in America during the 2009–2010 influenza season (21.8%) [20], and an H1N1/09 outbreak, with a seroprevalence (55%), occurred in a cat colony in Italy in November 2009 [15]. Zhao et al. found a particularly high seropositivity rate for H1N1/09 (21%) in northeastern China from 2012 to 2013 [21]. Our results show that the highest seropositivity rate in cats during the period examined was in 2010, which was possibly related to the outbreak of the H1N1/09 virus in humans in China.

The present study demonstrates that the H1N1/09, canine H3N2, and human H3N2 influenza viruses are prevalent in cats in China. Therefore, the continuous surveillance of influenza viruses in cats in China is necessary to monitor the threat of infection at the human–animal interface.
